# Exploring the Design of Upper Limb Strength Training Through High-Intensity Interval Training Combined With Exergaming: Usability Study

**DOI:** 10.2196/51730

**Published:** 2024-04-17

**Authors:** Shu-Cheng Lin, Jing-Yu Lee, Yong Yang, Chu-Chun Fang, Hsiao-Lin Fang, Tien-Hung Hou

**Affiliations:** 1 Department of Sport, Leisure and Health Management, Tainan University of Technology, Tainan City, Taiwan; 2 Laboratory of Kinesiology and Rehabilitation, School of Physical Education and Sport, Chaohu University, Hefei, China; 3 National Taipei University of Business Physical Education Office, Taipei City, Taiwan; 4 Department of Styling & Cosmetology, Tainan University of Technology, Tainan City, Taiwan; 5 General Education Center and Regimen and Leisure Management (Jointly Appointed), Tainan University of Technology, Tainan City, Taiwan; 6 Sustainable Environment and Technology Application Research Center, Tainan University of Technology, Tainan City, Taiwan

**Keywords:** muscle, electromyography, healthy, home training, exercise

## Abstract

**Background:**

High-intensity interval training (HIIT) has become a popular exercise strategy in modern society, with the Tabata training method being the most popular. In the past, these training methods were mostly done without equipment, but incorporating exergaming into the training may provide a new option for muscle training.

**Objectives:**

The aim of this study was to explore the differences in upper limb muscle activation using an HIIT program combined with exergaming.

**Methods:**

A total of 15 healthy male participants were recruited for the study, and the differences in muscle activation were compared between push-ups and exergaming (Nintendo Switch Ring Fit Adventure with the Ring-Con accessory) during HIIT. Prior to the tests, participants underwent pretests, including maximal voluntary contractions of various muscle groups, maximal push-up tests, and maximal movement tests using the exergaming device. The push-up and exergaming tests were conducted on separate days to avoid interference, with a warm-up period of 5 minutes on a treadmill before testing. Muscle activation in the lateral and anterior portions of the deltoid muscle, the sternal and clavicular heads of the pectoralis major muscle, and the latissimus dorsi muscle were measured during the maximal voluntary contractions and single-round tests for each exercise mode. A repeated measures ANOVA was used to assess the variations in muscle activation observed across the 2 distinct modes of exercise, specifically push-ups and exergaming.

**Results:**

In exergaming, the number of repetitions for push-ups was significantly fewer than for single-site exercises across both exhaustive (mean 23.13, SD 6.36 vs mean 55.67, SD 17.83; *P*=.001; effect size [ES]: 2.43) and single-round (mean 21.93, SD 7.67 vs mean 92.40, SD 20.47; *P*=.001; ES: 4.56) training. Heart rate differences were not significant (all *P*>.05), yet exergaming led to better muscle activation in specific muscle groups, particularly the right anterior deltoid (mean 48.00%, SD 7.66% vs mean 32.84%, SD 10.27%; *P*=.001; ES: 1.67) and right pectoralis major (sternal head: mean 38.99%, SD 9.98% vs mean 26.90%, SD 12.97%; *P*=.001; ES: 1.04; clavicular head: mean 43.54%, SD 9.59% vs mean 30.09%, SD 11.59%; *P*=.002; ES: 1.26) during exhaustive training. In single-round training, similar patterns were observed with the anterior deltoid (mean 51.37%, SD 11.76% vs mean 35.47%, SD 12.72%; *P*=.002; ES: 1.30) and pectoralis major (sternal head: mean 53.27%, SD 10.79% vs mean 31.56%, SD 16.92%; *P*=.001; ES: 1.53; clavicular head: mean 53.75%, SD 13.01% vs mean 37.95%, SD 14.67%; *P*=.006; ES: 1.14). These results suggest that exergaming may be more effective for targeted muscle activation.

**Conclusions:**

In conclusion, HIIT can increase muscle activation in the upper extremities and can be incorporated into exergaming strategies to provide a fun and engaging way to exercise.

## Introduction

In recent years, motion-based video games have made substantial contributions to both medical education and sports training [1]. They have shown notable effects in the rehabilitation or training of upper limbs [2-4]. The enjoyment derived from gaming can enhance participants’ motivation, and when combined with specific game design, it becomes one of the hot topics in research. Compared to longer-duration, moderate-intensity exercise, the strategy of high-intensity interval training (HIIT) [5] has become the mainstream exercise approach in modern society. The most popular approach is the Tabata training method, which involves performing 8 cycles of 20 seconds of all-out exercise, interspersed with 10 seconds of complete rest, for a total exercise time of 240 seconds [6]. Results have shown significant improvements in aerobic power [7], fat oxidation [8], and muscular endurance [9]. It can be observed that the HIIT strategy not only shortens exercise participation time but also has positive effects on the body. Tabata exercises, apart from running, also include various forms of bodyweight exercises, such as push-ups, squats, and burpees [10]. Among these, push-ups are the most used bodyweight exercise in Tabata training. In a study on muscle activation for strength training, Alizadeh et al [11] investigated the muscle activation patterns of push-ups and sit-ups, measuring the activation of the anterior and lateral portions of the deltoid muscle, as well as the sternal and clavicular heads of the pectoralis major muscle. The results showed variations in muscle activation levels despite the similarity in the exercises, highlighting differences in muscle engagement across different parts of the body. Another study by Putra et al [12] explored the muscle activation levels in the upper limbs during a boxing game while in standing and sitting positions in virtual reality gaming. The study found significant differences in the activation of the upper trapezius muscle during uppercut punches, whereas no differences were observed in straight and hook punches. Combining the findings of these 2 studies, it is evident that different exercises lead to varying levels of muscle activation in different muscle groups.

Push-ups, historically used to assess upper body strength, are frequently incorporated into HIIT sessions. This exercise primarily targets the deltoid, pectoralis major, and latissimus dorsi muscles. Similarly, virtual reality gaming offers training modes specifically designed to target these muscle groups. In summary, the research highlights the diverse muscle activation patterns associated with different exercises. Push-ups, a fundamental bodyweight exercise, have been traditionally used to assess upper body strength and are a common component of HIIT workouts, effectively engaging the deltoid, pectoralis major, and latissimus dorsi muscles. Additionally, virtual reality gaming provides tailored training modes focusing on these specific muscle groups. For individuals engaged in recreational physical activities, these conventional exercise methods might be perceived as monotonous due to their limited variation, potentially leading to reduced adherence to training. This lack of variety could negatively impact exercise adherence, as the “lack of enjoyment” is frequently cited as a common barrier to regular physical activity [13].

In studies on the application of HIIT in strength training, the focus has been on investigating the rest intervals between exercises [14] and movement speed [15]. Tomoo et al [14] examined the effects of long and short rest intervals between sets and found that shorter rest intervals led to higher muscle activation levels at the same exercise intensity. In contrast, Dora et al [15] found that faster movement speed resulted in higher muscle activation levels. Taken together, these studies suggest that shorter rest intervals and faster movement speed lead to greater muscle activation. Previous literature has also shown that shorter rest intervals can improve muscle adaptations during resistance training [16,17]. However, in upper body exercise design, push-ups are commonly included as one of the training movements and are frequently used to assess upper body muscle strength and endurance [18].

Exergaming have been used for exercise training for many years and have contributed to improving exercise participation [19]. Related exergaming devices includes Xbox 360, Nintendo Wii, Nintendo Switch, and Sony PlayStation 2. Through exergaming, aerobic capacity, agility, muscle strength, muscle endurance, and coordination can be improved [20]. Regarding muscle strength training, Willaert et al [21] showed that muscle activation can be improved by more than 40%. Although the level of activation is relatively low, this study aims to design a training program with the combination of HIIT and body-sensing video games to enhance the quality and effectiveness of the training. There are relatively few studies on the use of HIIT for muscle strength training, and the level of muscle activation using exergaming combined with HIIT has not been clarified. The aim of this study was to explore the differences in upper limb muscle activation using an HIIT program combined with exergaming.

## Methods

### Study Participants

In this study, we recruited 15 healthy male participants. They had an average age of 24.4 (SD 10.4) years, stood at an average height of 174 cm with a minimal variance of 0.05 cm, and weighed an average of 71.9 (SD 13) kg. Their BMI averaged at 23.5 (SD 3.57) kg/m^2^. All participants maintained a regular exercise routine, engaging in physical activity 3 times per week over the past year, and had experience in performing push-ups correctly. They also completed the Physical Activity Readiness Questionnaire [22] and confirmed that they had no history of upper or lower limb skeletal muscle injury or major injury. Participants were instructed to avoid vigorous activity and the intake of caffeine or supplements that enhance muscle performance for 24 hours prior to the experiment. Before the study began, all participants provided their personal information and medical history and filled out the health questionnaires and informed consent form. Additionally, the data were proofread to ensure accuracy and readability.

### Ethical Considerations

The human research ethics committee of the local university approved this study, which was also approved by the human research ethics committee of the National Cheng Kung University, Taiwan (approval NCKU HREC-E-112-419-2). Users volunteered for this study and agreed to participate by signing an informed consent form. The research ensures the issues of privacy and confidentiality by assigning participants with numerical identifiers during the experiment to safeguard the confidentiality of their personal information. In terms of compensation, participants were volunteers and there was no remuneration involved.

### Experimental Design

#### Overview

**Figure 1.**
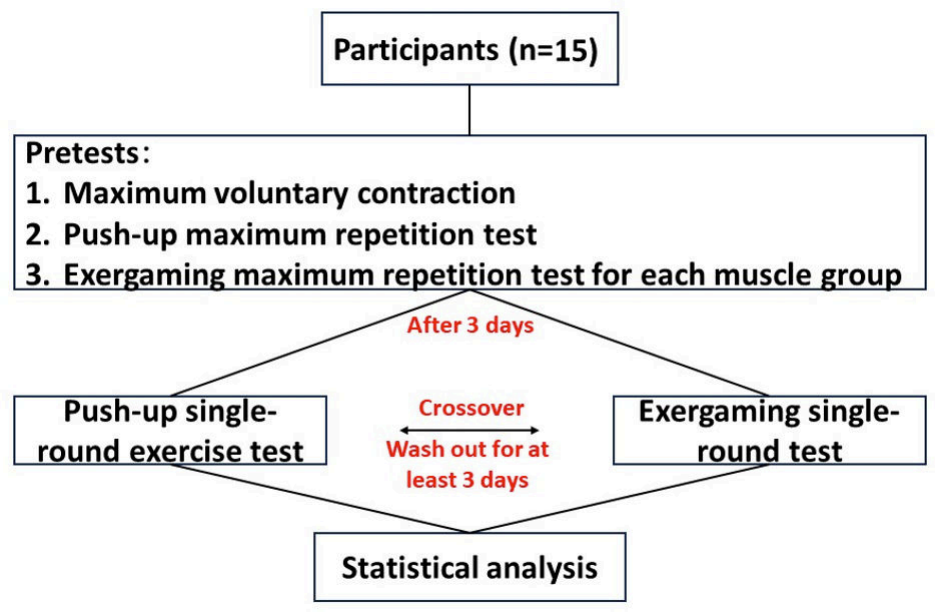
Experimental procedure diagram.

This study used a randomized, crossover, and repeated measures experimental design to compare the differences in muscle activation between push-up exercise and a Nintendo Switch Ring Fit Adventure exercise. Prior to the tests, participants underwent pretests, which included maximal voluntary contractions (MVCs) for each muscle group, maximal push-up tests, and maximal exercise tests for each part of the Nintendo Switch Ring Fit Adventure exercise. The 2 types of tests were conducted with a minimum interval of 3 days to avoid interference. Prior to each test, a 5-minute warm-up on the treadmill at a speed of 2 m/s was recommended. During the tests, muscle activation in various muscle groups was observed and measured, including the lateral and anterior portions of the deltoid muscle, the sternal and clavicular heads of the pectoralis major muscle, and the latissimus dorsi muscle, which were referenced and modified from previous studies by van den Tillaar [23], Alizadeh et al [11], and Maeo et al [24] (Figure 1). The aim was to explore the differences in upper limb muscle activation using an HIIT program combined with exergaming.

#### Exergaming

**Figure 2.**
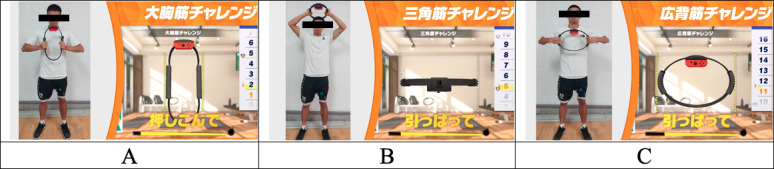
Exercise training and exergaming model: (A) pectoralis major, (B) deltoid, and (C) latissimus dorsi.

The exergaming selected for this study was Nintendo Switch Ring Fit Adventure, which combines exercise, adventure, and entertainment, allowing players to enjoy both physical workouts and gaming fun simultaneously. The game features an intuitive and user-friendly interface, suitable for players of all ages. It comes with a specialized fitness ring device (Ring-Con), an intelligent accessory that connects to the Nintendo Switch console. Through this ring, players can engage in various physical activities such as weightlifting, yoga, and aerobic exercises. The fitness ring sensor accurately captures players’ movements and incorporates them into the gameplay. The game content involves unlocking levels and participating in fitness competitions through real-life physical movements. It offers a variety of fitness activities, each targeting different muscle groups, while also providing enjoyable gaming challenges. The trained muscle groups were the pectoralis major, deltoid, and latissimus dorsi muscles. The testing consisted of 2 modes: (1) maximum repetition test and (2) single-round test. The maximum repetition test was conducted using the extreme challenge mode. Participants were instructed to follow the game’s pace of 60 beats per minute as closely as possible and maintain proper posture during each muscle group’s testing to avoid compensation from other muscles. The single-round test was conducted using the challenge mode. Participants were instructed to perform repetitive actions as quickly as possible for 20 seconds (Figure 2).

#### Push-Up Tests

The push-up tests included 2 types of tests: (1) maximum repetition test and (2) single-round test. The maximum repetition test was based on the testing method described by Eckel et al [25]. Participants were guided to execute the test while in sync with a metronome set at 60 beats per minute, ensuring each movement, 1 second downward and 1 second upward, matched the rhythm precisely. This cadence was chosen to align with the pace of exercises conducted in the Nintendo Switch Ring Fit Adventure exercise, facilitating a consistent and controlled environment for comparison. During the test, the distance between the hands at the sternal notch level was measured and must be the same as the distance used in the single-round test. The elbow must be bent at 90° and the elbow must be fully extended when straightened. The single-round test was designed to match the duration of a single round of the Nintendo Switch Ring Fit Adventure exercise and involved performing the maximum number of squats possible within 20 seconds.

#### Heart Rate Tests

Heart rate measurements were taken using the iHeart heart rate sensor (Hexin) during push-up tests and exergaming sessions. The heart rate sensor was worn directly below the sternum and in direct contact with the skin, ensuring a comfortable fit that remained secure without slipping, even during exercise. After being fitted, the sensor was connected to the Polar Beat app (Polar Electro) for monitoring and recording purposes.

### Electromyography

#### MVC Testing

**Figure 3.**
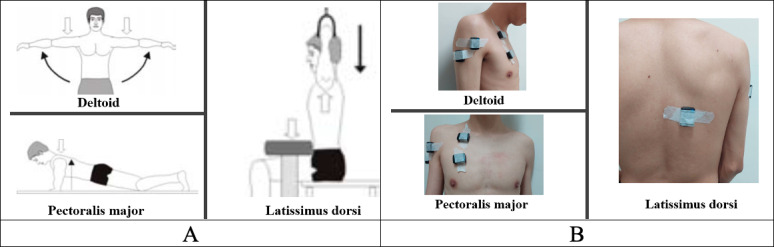
(A) Standardized illustrations for the MVC action of each muscle. (B) MVC sensor placement for each muscle. MVC: maximal voluntary contraction.

In MVC testing, each muscle group underwent an isometric MVC prior to the test. After a running warm-up and a 3-minute rest period, the MVC test was performed according to the movements described by Konrad [26]. The test consisted of 3 attempts, each lasting 5 seconds with 1-minute rest intervals in between, and the participant was encouraged to exert maximum effort during each attempt. If the peak MVC values differed by more than 5%, additional testing was performed. The test procedures for each muscle group were as follows. (1) Deltoid: the participants were seated with their backs supported, and their arms were abducted to a position where they formed a 90° angle with the trunk, maintaining a horizontal plane. A rope, fixed to the arm, was then pulled upward to measure the force exerted. (2) Pectoralis major: the participants performed the test in a lying position with the elbows extended and flexed at 90°, holding onto a long bar with a fixed rope attached to each side of the bar and pulling upward as hard as possible to measure the force exerted. (3) Latissimus dorsi: the participants performed the test in a seated position, simulating the movement of a pull-up. The axis joint was flexed and abducted at 90°, and the rope was pulled downward the measure the force exerted, as shown in Figure 3. After the MVC testing, electromyography (EMG) electrodes were placed on the skin over the belly of the tested muscles for subsequent signal recording and analysis.

#### EMG Measurement and Analysis

The Trigno TM wireless foundation system (Delsys-EMGworks) was used for data collection in this study, measuring the anterior and lateral portions of the deltoid muscle, the sternal and clavicular heads of the pectoralis major muscle, and the latissimus dorsi muscle [11,23,24]. The system was configured with a sampling rate set at 2000 Hz per channel, tailored to the desired sample rate specifications. For the processing of EMG data, we used the EMGwork analysis software, which included steps of filtering and smoothing the EMG signals to ensure clarity and accuracy. The filtering process used a band-pass filter with a low-frequency cutoff at 20 Hz and a high-frequency cutoff at 500 Hz. Following this, the rectified EMG signals underwent further refinement using the root mean square method, which facilitated a detailed analysis of the signal’s magnitude. During the MVC test, we determined the highest EMG amplitude recorded for each muscle group, referred to as EMGmax. The data processing method in exergaming was the same as the above, and the degree of muscle activation was calculated based on the values obtained from the standardized action test, expressed as a percentage of EMGmax.

### Statistical Analysis

Data processing and analysis were performed using SPSS for Windows (version 20.0; IBM Corp). The data were presented as mean and SD. To investigate the variance in motion and heart rate across different movements derived from the 2 exercise models—strength endurance and single round—we used a repeated measures ANOVA. Furthermore, to assess the discrepancies in muscle activation elicited by the 2 distinct types of exercises, namely push-ups and exergaming, a paired-samples 2-tailed *t* test was used. Cohen *d* for effect size (ES) was calculated by the G*Power 3.1 software program (Heinrich-Heine-Universität), where the ESs of 0.2, 0.5, and 0.8 were considered small, medium, and large, respectively. Statistical significance was set as *P*<.05.

## Results

### Study Participants

For this study, a total of 15 male participants from the community were recruited. These participants were generally in good health. However, finding healthy female participants capable of performing push-ups was challenging due to their limited availability. Therefore, this study predominantly concentrated on male participants.

### Comparing Strength, Performance, and Heart Rate: Push-Ups Versus Exergaming

The results showed that regardless of the exhaustive or single-round mode, the number of single-site repetitions in exergaming was significantly higher than that of push-ups (exhaustive: deltoid, mean 55.67, SD17.83; pectoralis major, mean 52.53, SD 13.61; and latissimus dorsi, mean 82.30, SD 20.82 vs push-up, 23.13, SD 6.36; *P*=.001; ES: 2.43, 2.77, and 3.84, respectively; single round: deltoid, mean 92.40, SD 20.47; pectoralis major, mean 104.27, SD 13.48; and latissimus dorsi, mean 97.33, SD 16.77 vs push-ups mean 21.93, SD 7.67; *P*=.001; ES: 4.56, 7.51, and 5.78, respectively). However, there was no difference in heart rate between the 2 modes (all *P*>.05). Taken together, these results suggest that both whole-body push-ups and single-site exergaming training can increase heart rate and can be used to train cardiorespiratory fitness (Table 1).

**Table 1.** Comparison of strength endurance, single-round performance, and heart rate between push-ups and exergaming.

Model and motion		Repetitions, mean (SD)	*F* test (*df*)	*P* value	Heart rate (bpm^a^), mean (SD)	*F* test (*df*)	*P* value
**Strength endurance test**			50.83	.001		3.92	.07
	Push-up	23.13 (06.36)			101.27 (14.79)		
	Exergaming, deltoid	55.67 (17.83)			96.80 (21.29)		
	Exergaming, pectoralis major	52.53 (13.61)			98.06 (18.76)		
	Exergaming, latissimus dorsi	82.30 (20.82)			91.40 (14.23)		
**Single-round performance (20 s)**			143.27	.001		1.56	.21
	Push-up	21.93 (07.67)			100.33 (16.32)		
	Exergaming, deltoid	92.40 (20.47)			104.80 (14.63)		
	Exergaming, pectoralis major	104.27 (13.48)			93.67 (17.11)		
	Exergaming, latissimus dorsi	97.33 (16.77)			104.13 (16.05)		

a bpm: beats per minute.

### Strength Endurance: Push-Ups Versus Exergaming

According to the statistical results, in the exhaustive mode, the activation levels of the right anterior deltoid (mean 48.00%, SD 7.66% vs mean 32.84%, SD 10.27%; *P*=.001; ES: 1.67), right pectoralis major—sternal head (mean 38.99%, SD 9.98% vs mean 26.90%, SD 12.97%; *P*=.001; ES: 1.04), and right pectoralis major—clavicular head (mean 43.54%, SD 9.59% vs mean 30.09%, SD 11.59%; *P*=.002; ES:1.26) were significantly greater in the exergaming group than in the push-up group. Thus, these results suggest that exergaming have a better training effect on specific muscle groups (Table 2).

**Table 2.** Comparison of muscle activation between traditional push-ups and exergaming in the strength endurance test.

Muscle	Push-up activation (%), mean (SD)	Exergaming activation (%), mean (SD)	*t* test (*df*)	*P* value
Anterior deltoid	32.84 (10.27)	48.00 (7.66)	−4.096	.001
Lateral deltoid	37.36 (13.79)	44.56 (8.82)	−1.737	.10
Pectoralis major, sternal head	26.90 (12.97)	38.99 (9.98)	−4.358	.001
Pectoralis major, clavicular head	30.09 (11.59)	43.54 (9.59)	−3.784	.002
Latissimus dorsi	35.43 (10.39)	34.01 (18.05)	0.329	.75

### Single-Round Test: Push-Ups Versus Exergaming

Based on the statistical analysis, in the single-round mode, the activation levels of the right anterior deltoid (mean 51.37%, SD 11.76% vs mean 35.47%, SD 12.72%; *P*=.002; ES: 1.30), right lateral deltoid (mean 52.08%, SD 10.79% vs mean 43.86%, SD 10.48%; *P*=.046; ES: 0.77), right pectoralis major–sternum head (mean 53.27%, SD 10.79% vs mean 31.56%, SD 16.92%; *P*=.001; ES: 1.53), and right pectoralis major–clavicle head (mean 53.75%, SD 13.01% vs mean 37.95%, SD 14.67%; *P*=.006; ES: 1.14) were significantly greater in the exergaming group than the push-up group. These results suggest that the exergaming designed as an HIIT exercise targeting a specific muscle group resulted in significantly higher muscle activation compared to push-ups (Table 3).

**Table 3.** Comparison of muscle activation between traditional push-ups and exergaming in the single-round test.

Muscle	Push-up activation (%), mean (SD)	Exergaming activation (%), mean (SD)	*t* test (*df*)	*P* value
Anterior deltoid	35.47 (12.72)	51.37 (11.76)	−3.705	.002
Lateral deltoid	43.86 (10.48)	52.08 (10.79)	−2.190	.046
Pectoralis major, sternal head	31.56 (16.92)	53.27 (10.79)	−4.266	.001
Pectoralis major, clavicular head	37.95 (14.67)	53.75 (13.01)	−3.236	.006
Latissimus dorsi	41.49 (11.00)	38.17 (16.87)	0.645	.529

## Discussion

### Principal Findings

The results indicated that, in terms of the number of repetitions performed, both the strength endurance and single-round tests showed significantly higher execution rates for the exergaming training mode compared to push-ups. However, there were no significant differences in heart rate between the 2 modes. Regarding muscle activation, in the strength endurance test, exergaming exhibited significantly higher activation levels than push-ups in the anterior deltoid, pectoralis major–sternal head, and pectoralis major–clavicular head muscles. In the single-round test, exergaming demonstrated significantly higher activation levels than push-ups in the anterior deltoid, lateral deltoid, pectoralis major–sternal head, and pectoralis major–clavicular head muscles.

### Heart Rate Response in Exergaming

Interestingly, the study did not observe significant differences in heart rate between the exergaming and push-up groups. This result contrasts with previous studies indicating that exergaming can lead to higher heart rates due to the immersive and stimulating nature of video game–based exercises [27,28]. The lack of significant heart rate differences could be attributed to the individual variability in cardiovascular responses and the adaptability of participants to the exergaming interface. HIIT has been shown to be an effective way to improve cardiovascular fitness and overall health [7]. Heart rate is an important factor in both exergaming and HIIT. Monitoring heart rate can help individuals ensure that they are working at an appropriate intensity level to achieve their fitness goals. In HIIT, heart rate can be used to guide the high-intensity intervals and rest periods to optimize the workout’s effectiveness [29]. Overall, the use of exergaming and HIIT can provide a fun and effective way to improve physical fitness and health, with heart rate monitoring serving as an important tool to help individuals achieve their goals.

Another potential explanation for these results is that the use of exergaming may increase motivation and engagement in physical activity, leading to greater adherence to exercise programs [30]. This is especially important given the high rates of sedentary behavior and physical inactivity in modern society. Exergaming may provide a fun and enjoyable way to engage in physical activity, potentially leading to increased frequency and duration of exercise sessions [31,32]. This is particularly relevant for individuals who may struggle to engage in more traditional forms of exercise due to boredom, lack of motivation, or physical limitations.

### EMG Response in Exergaming

This study investigated the effects of HIIT on muscle activation in the upper extremities. The results suggest that exergaming may be a more effective training method for upper extremity muscle activation compared to push-up exercises. The reason for this difference may be due to the specific muscles activated during exergaming, as the Nintendo Switch Ring-Con requires movements that engage the lateral and anterior parts of the deltoid muscle, pectoralis major muscle, and latissimus dorsi muscle. These findings suggest that exergaming can be a viable option for those looking to improve upper extremity muscle activation.

In interpreting the results, the study found that the exergaming training mode exhibited superior performance in terms of the number of repetitions compared to traditional push-ups, both in the strength endurance test and the single-round test. This finding aligns with previous research indicating the effectiveness of exergaming in enhancing endurance and strength capacities [33,34]. The higher execution rates in the exergaming group suggest that this interactive gaming approach offers a more engaging and motivating environment, encouraging participants to perform better and prolong their workout sessions compared to conventional push-ups. EMG can reflect the response of muscles during strength training. Alizadeh et al [11] investigated 2 common strength training exercises, push-ups and sit-ups, and measured muscle activation in the major muscle groups involved, such as the lateral and anterior portions of the deltoid and the sternal and clavicular heads of the pectoralis major. The results showed that even with the same exercise, different muscle groups were activated to varying degrees, indicating the importance of focusing on specific muscle group activation for muscle training. Compared to traditional strength training, there has been relatively little research on exergaming, but Putra et al [12] investigated the activation levels of upper limb muscles during a punching game while in standing and sitting positions. The results showed significant differences in the activation of the upper trapezius muscle during the execution of an uppercut punch, but not for straight or hook punches, indicating that the fixedness of the movements also affects the activation levels of different muscle groups in exergaming. Taken together, these studies suggest that different movements can affect muscle activation levels in different muscle groups. Therefore, choosing appropriate exercises and training modes is important for muscle strength training. Push-ups are a common exercise that mainly trains muscle groups, such as the deltoid, pectoralis major, and latissimus dorsi muscles, and are commonly used in HIIT. Training modes in exergaming are also available for these muscle groups, providing more diverse options for muscle training. Overall, these research results indicate the importance of understanding the relationship between movements and muscle groups.

Regarding muscle activation patterns, the exergaming group demonstrated significantly higher activation levels in specific muscles compared to traditional push-ups. In the strength endurance test, the anterior deltoid, pectoralis major–sternal head, and pectoralis major–clavicular head muscles exhibited increased activation during exergaming sessions. These findings corroborate with prior studies highlighting the targeted muscle engagement achieved through exergaming interventions [21]. The single-round test further showed elevated muscle activation in the anterior deltoid, lateral deltoid, pectoralis major–sternal head, and pectoralis major–clavicular head muscles during exergaming activities. This specific muscle activation pattern emphasizes the comprehensive nature of exergaming exercises, engaging various upper body muscles simultaneously [35]. One potential explanation for these results is that the Ring-Con is a novel type of resistance training that provides a more targeted and isolated workout for specific muscle groups [36]. This may allow individuals to activate and recruit more muscle fibers, leading to increased muscle activation compared to more traditional exercises such as push-ups. Additionally, the Ring-Con provides a unique form of resistance that can be adjusted to individual fitness levels, potentially allowing for greater customization and variety in workout routines [12].

### Conclusions

In conclusion, this study demonstrated that exergaming may be a more effective strategy for upper extremity muscle activation compared to push-up exercises during HIIT. The specific movements required by the Nintendo Switch Ring-Con may activate the lateral and anterior parts of the deltoid muscle, pectoralis major muscle, and latissimus dorsi muscle more effectively. Furthermore, the HIIT protocol used in this study provides a time-efficient method for strength training. Incorporating exergaming into an HIIT program may provide a more engaging and effective strategy for improving upper extremity muscle activation. Further research is needed to investigate the long-term effects of exergaming on upper extremity muscle activation and strength.

## Abbreviations

EMG: electromyography
ES: effect size
HIIT: high-intensity interval training
MVC: maximal voluntary contraction

## References

[1] Tolks D, Schmidt JJ, Kuhn S (2024). The role of AI in serious games and gamification for health. JMIR Serious Games.

[2] Koutsiana E, Ladakis I, Fotopoulos D, Chytas A, Kilintzis V, Chouvarda I (2020). Serious gaming technology in upper extremity rehabilitation: scoping review. JMIR Serious Games.

[3] Vieira C, Ferreira da Silva Pais-Vieira C, Novais J, Perrotta A (2021). Serious game design and clinical improvement in physical rehabilitation: systematic review. JMIR Serious Games.

[4] Villada Castillo JF, Montoya Vega MF, Muñoz Cardona JE (2024). Design of virtual reality exergames for upper limb stroke rehabilitation following iterative design methods: usability study. JMIR Serious Games.

[5] Weston M, Taylor KL, Batterham AM, Hopkins WG (2014). Effects of low-volume high-intensity interval training (HIT) on fitness in adults: a meta-analysis of controlled and non-controlled trials. Sports Med.

[6] Viana RB, de Lira CAB, Naves JPA, Coswig VS, del Vecchio FB, Gentil P (2019). Tabata protocol: a review of its application, variations and outcomes. Clin Physiol Funct Imaging.

[7] Viana RB, Naves JPA, de Lira CAB (2018). Defining the number of bouts and oxygen uptake during the "Tabata protocol" performed at different intensities. Physiol Behav.

[8] Pearson RC, Olenick AA, Green ES, Jenkins NT (2020). Tabata‐style functional exercise increases resting and postprandial fat oxidation but does not reduce triglyceride concentrations. Exp Physiol.

[9] Menz V, Marterer N, Amin SB, Faulhaber M, Hansen AB, Lawley JS (2019). Functional vs running low-volume high-intensity interval training: effects on VO2max and muscular endurance. J Sports Sci Med.

[10] Tabata I (2019). Tabata training: one of the most energetically effective high-intensity intermittent training methods. J Physiol Sci.

[11] Alizadeh S, Rayner M, Mahmoud MMI, Behm DG (2020). Push-ups vs bench press differences in repetitions and muscle activation between sexes. J Sports Sci Med.

[12] Putra BAM, Masduchi RH, Tinduh D, Pawana I (2021). Upper limb muscles activity during punches in virtual reality exergame on standing and sitting position. Surabaya Physical Medicine and Rehabilitation Journal.

[13] Bartlett JD, Close GL, MacLaren DPM, Gregson W, Drust B, Morton JP (2011). High-intensity interval running is perceived to be more enjoyable than moderate-intensity continuous exercise: implications for exercise adherence. J Sports Sci.

[14] Tomoo K, Suga T, Dora K (2021). Impact of inter-set short rest interval length on inhibitory control improvements following low-intensity resistance exercise in healthy young males. Front Physiol.

[15] Dora K, Suga T, Tomoo K (2021). Effect of very low-intensity resistance exercise with slow movement and tonic force generation on post-exercise inhibitory control. Heliyon.

[16] Hughes L, Paton B, Rosenblatt B, Gissane C, Patterson SD (2017). Blood flow restriction training in clinical musculoskeletal rehabilitation: a systematic review and meta-analysis. Br J Sports Med.

[17] Goto K, Nagasawa M, Yanagisawa O, Kizuka T, Ishii N, Takamatsu K (2004). Muscular adaptations to combinations of high- and low-intensity resistance exercises. J Strength Cond Res.

[18] van den Tillaar R, Saeterbakken A (2014). Effect of fatigue upon performance and electromyographic activity in 6-RM bench press. J Hum Kinet.

[19] Bock BC, Dunsiger SI, Ciccolo JT (2019). Mediators of physical activity between standard exercise and exercise video games. Health Psychol.

[20] Comeras-Chueca C, Marin-Puyalto J, Matute-Llorente A, Vicente-Rodriguez G, Casajus JA, Gonzalez-Aguero A (2021). Effects of active video games on health-related physical fitness and motor competence in children and adolescents with overweight or obesity: systematic review and meta-analysis. JMIR Serious Games.

[21] Willaert J, de Vries AW, Tavernier J, van Dieen JH, Jonkers I, Verschueren S (2020). Does a novel exergame challenge balance and activate muscles more than existing off-the-shelf exergames?. J Neuroeng Rehabil.

[22] Adams R (1999). Revised Physical Activity Readiness Questionnaire. Can Fam Physician.

[23] van den Tillaar R (2019). Comparison of kinematics and muscle activation between push-up and bench press. Sports Med Int Open.

[24] Maeo S, Chou T, Yamamoto M, Kanehisa H (2014). Muscular activities during sling- and ground-based push-up exercise. BMC Res Notes.

[25] Eckel TL, Watkins CM, Archer DC (2017). Bench press and pushup repetitions to failure with equated load. International Journal of Sports Science & Coaching.

[26] Konrad P (2005). The ABC of EMG: A Practical Introduction to Kinesiological Electromyography.

[27] Kircher E, Ketelhut S, Ketelhut K (2022). Acute effects of heart rate-controlled exergaming on vascular function in young adults. Games Health J.

[28] Kraft JA, Russell WD, Bowman TA, Selsor CW III, Foster GD (2011). Heart rate and perceived exertion during self-selected intensities for exergaming compared to traditional exercise in college-age participants. J Strength Cond Res.

[29] Ito S (2019). High-intensity interval training for health benefits and care of cardiac diseases - the key to an efficient exercise protocol. World J Cardiol.

[30] Sadeghi H, Jehu DA, Daneshjoo A (2021). Effects of 8 weeks of balance training, virtual reality training, and combined exercise on lower limb muscle strength, balance, and functional mobility among older men: a randomized controlled trial. Sports Health.

[31] Sween J, Wallington SF, Sheppard V, Taylor T, Llanos AA, Adams-Campbell LL (2014). The role of exergaming in improving physical activity: a review. J Phys Act Health.

[32] Vandoni M, Codella R, Pippi R (2021). Combatting sedentary behaviors by delivering remote physical exercise in children and adolescents with obesity in the COVID-19 era: a narrative review. Nutrients.

[33] Viana RB, de Oliveira VN, Dankel SJ (2021). The effects of exergames on muscle strength: a systematic review and meta‐analysis. Scand Med Sci Sports.

[34] Esmaeilzadeh S, Kumpulainen S, Pesola AJ (2022). Strength-cognitive training: a systemic review in adults and older adults, and guidelines to promote “strength exergaming” innovations. Front Psychol.

[35] Novák D (2015). Handbook of Research on Holistic Perspectives in Gamification for Clinical Practice.

[36] Owen PJ, Miller CT, Mundell NL (2020). Which specific modes of exercise training are most effective for treating low back pain? network meta-analysis. Br J Sports Med.

[37] ChatGPT OpenAI. https://chat.openai.com/.

